# Erratum: Cutaneous rabbit “hops toward a light: unimodal and cross-modal causality on the skin”

**DOI:** 10.3389/fpsyg.2013.00769

**Published:** 2013-10-18

**Authors:** Tomohisa Asai, Noriaki Kanayama

**Affiliations:** ^1^Department of Psychology, Chiba University, Chiba-shi, Japan; ^2^Department of Cognitive and Behavioral Science, Graduate School of Arts and Sciences, The University of Tokyo, Tokyo, Japan

**Keywords:** cutanous rabbit effect, multi-modal integration, vision, tactile, localization

We would like to apply corrections regarding word-by-word citations.

**Abstract**

**The following sentences**

Repeated rapid stimulation at the wrist, then near the elbow, can create the illusion of touch at intervening locations along the arm (as if a rabbit is hopping along the arm).

**->should be replaced by**

Under some conditions, successive tactile stimuli at some locations on the forearm can be felt as an illusion of movement along the arm (as if a rabbit is hopping along the arm).

**P1, left column**

L6

A percept that misrepresents physical reality (i.e., an illusion) is both a consequence of and a clue as to the brain's expectations regarding the external world (Goldreich, 2007).

->

We can know through some perceptual illusions how the brain expects an event that is happening in the external world (Goldreich, 2007).

L9

The brain takes advantage of prior knowledge to enhance its perceptual resolution. In the case of tactile perception, spatial imprecision due to low receptor density poses a particular challenge (Goldreich, 2007).

->

The brain can utilize prior knowledge to overcome its limitation in perceptual resolution. For example, the spatial resolution in tactile perception depends on its low density of receptors (Goldreich, 2007).

L12

Even without the benefit of exploratory movements, the fingertips' resolving power- the most discriminating tactile sensor among primates - is on the order of 1 mm (Weinstein, 1968; Johnson and Phillips, 1981).

->

The spatial tactile resolution at the fingertips, where primates have the most discriminating tactile sensor, is on the order of 1 mm (Weinstein, 1968; Johnson and Phillips, 1981).

L17

This is the case even though the brain contains a representation of the body map in the primary somatosensory cortex (S1; Penfield and Boldrey, 1937) which reflects the locations of physical stimuli on the skin.

->

This is the case even though the brain contains a body representation or so-called body map in the primary somatosensory cortex (S1; Penfield and Boldrey, 1937), which reflects the locations of physical stimuli on the skin.

L20

Furthermore, given the several-ms jitter in the stimulus-evoked - first-spike latencies of somatosensory cortical neurons (Foffani et al., 2004), the somatosensory system has not only spatial but also temporal imprecision.

->

Furthermore, given the stimulus-evoked first-spike latencies of somatosensory cortical neurons (Foffani et al., 2004), the somatosensory system has not only spatial but also temporal imprecision.

**P1, right column**

L3

The CRE is a subset of a larger class of tactile saltation illusions elicited when a mechanical stimulus is followed by similar stimuli rapidly applied at nearby locations (Geldard and Sherrick, 1972; Warren et al., 2010).

->

The CRE is a “subset of a larger class of tactile saltation illusions” that could be caused when a stimulus is followed by similar stimuli rapidly applied at adjacent locations (Geldard and Sherrick, 1972; Warren et al., 2010).

L6

For example, repeated, rapid stimulation at the wrist and then near the elbow can create the illusion of touch at intervening locations along the arm, as if a rabbit is hopping along the arm.

->

For example, repeated rapid stimulation at a location near the wrist and then at another location near the elbow could be felt as an illusion of movement along the arm, including illusory touch at the intervening location, as if a rabbit is hopping along the arm.

L9

The apparent location of each stimulus moves from the actual stimulation site toward the other stimulation sites in a predictable manner depending on factors such as stimulus location and frequency (e.g., Geldard and Sherrick, 1972; Kilgard and Merzenich, 1995; Cholewiak, 1999; Flach and Haggard, 2006).

->

The apparent location of each stimulus moves from the actually stimulated location toward the other location “in a predictable manner” (e.g., Geldard and Sherrick, 1972; Kilgard and Merzenich, 1995; Cholewiak, 1999; Flach and Haggard, 2006).

L14

The CRE is apparently related to the classic tau effect (Goldreich, 2007), in which the more rapidly traversed of two equal distances defined by three stimuli is perceived as being shorter (Helson, 1930).

->

The tau effect apparently shares its underlying mechanism with the CRE (Goldreich, 2007): when two equal distances by three stimuli are traversed rapidly, the distance could be perceived as being shorter (Helson, 1930).

L17

When stimulus timing is held constant, the perceived distance between two stimuli both underestimates and grows in proportion with the actual inter-stimulus distance (Marks et al., 1982; Cholewiak, 1999).

->

As long as stimulus timing is kept constant, the perceived distance between two stimuli gets longer or shorter depending on the actual inter-stimulus distance (Marks et al., 1982; Cholewiak, 1999).

L22

These effects have been explained on the basis of the hypothetical idea that the sensory system imputes uniform motion to discontinuous dynamic displays; therefore, there is an assumption of constant velocity motion (Jones and Huang, 1982).

->

These effects can be interpreted to mean that the sensory system would understand discontinuous dynamic displays as uniform motion; that is, an assumption of constant velocity motion (Jones and Huang, 1982).

**P2, left column**

L2

Also, a recent Bayesian perceptual model replicated the CRE by assuming that the brain expects tactile stimuli to move slowly (Goldreich, 2007) since we have evolved to detect the movement of external agents (Leslie, 1995).

->

In addition, a recent study has suggested that the CRE can be simulated by a Beyesian model based on the assumption that the brain expects the slow movements of tactile stimuli (Goldreich, 2007) since we have evolved to detect the movement of external agents (Leslie, 1995).

L10

Certain simple visual displays consisting of moving, 2-D, geometric shapes can give rise to percepts with high level properties, such as causality and animacy. This suggests that just as the visual system works to recover the physical structure of the world by inferring properties such as 3-D shapes, it also works to recover the causal and social structures of the world by inferring properties such as causality and animacy (Scholl and Tremoulet, 2000).

->

Even simple 2-D moving objects can give us some impressions with high-level properties including causality and animacy, suggesting that “just as the visual system works to recover the physical structure of the world by inferring properties such as 3-D shapes, it also works to recover the causal and social structures of the world by inferring properties such as causality and animacy” (Scholl and Tremoulet, 2000).

L20

Given that many natural events can be perceived via multiple sensory modalities, we typically have access to multiple features of those events across different senses (Vroomen and Keetels,2010).

->

Given that many natural events can be perceived via multiple sensory modalities, we can gain access to multiple features of those external events across modalities (Vroomen and Keetels,2010).

L26

The ability to combine information from multiple sensory modalities into a single, unified percept is a key element of organisms' abilities to interact with the external world (Stevenson et al., 2011).

->

We can combine multiple information across different senses into a unified percept, which is fundamentally required in order for us to interact with the external world (Stevenson et al., 2011)

L29

This process of perceptual fusion - the amalgamation of multiple sensory inputs into a perceptual gestalt - is highly dependent on the temporal synchrony of sensory inputs (Meredith et al., 1987; Bishop and Miller, 2009; Stevenson et al., 2011).

->

This perceptual binding or “the amalgamation of multiple sensory inputs into a perceptual gestalt” is highly related to the temporal synchrony among sensory inputs (Meredith et al., 1987; Bishop and Miller, 2009; Stevenson et al., 2011).

L35

The combination of crossmodal information by humans is highly consistent with an optimal Bayesian model of causal inference; this phenomenon is known as “cross-modal causality” (Goldreich, 2007; Beierholm et al., 2009; Schutz and Kubovy,2009).

->

The human's ability to combine crossmodal information could be explained by an optimal Bayesian model of causal inference, a phenomenon is known as “cross-modal causality” (Goldreich, 2007; Beierholm et al., 2009; Schutz and Kubovy,2009).

**P2, right column**

L6

Indeed, the tactile tau and kappa effects are also susceptible to cross-modal (visual or auditory) influences (Suto, 1952; Russo and Dellantonio, 1989).

->

Indeed, the tactile tau and kappa effects are also modulated under the cross-modal presentation visually or auditorily (Suto, 1952; Russo and Dellantonio, 1989).

L19

Another study also suggested that the illusory somatosensory percepts caused by the CRE can affect the primary somatosensory cortex at a location corresponding to the illusory percept (Blankenburg et al., 2006).

->

Another study also suggested that the illusory somatosensory percepts caused by the CRE can affect the location in primary somatosensory cortex corresponding to the illusory percept (Blankenburg et al., 2006).

**P3, left column**

L6

This could indicate that our brain is tuned to detect the movement of an external agent on the skin since an essential, evolutionarily stable feature of brain function is the detection of animate entities for survival (Schultz et al.,2005; Pratt et al.,2010).

->

This could indicate that our brain is tuned to detect the movement of an external agent on the skin, since for survival, “an essential evolutionarily stable feature of brain function is the detection of animate entities” (Schultz et al.,2005; Pratt et al.,2010).

L10

Furthermore, we argue that sensory events at a certain time point are influenced by future sensory events; this is referred to as “postdictive” sensation (Eagleman and Sejnowski, 2000).

->

Furthermore, we argue that future sensory events affect past sensory experiences; this is referred to as “postdictive” sensation (Eagleman and Sejnowski, 2000).

L16

They were recruited randomly from an introductory psychology class, and written informed consent was obtained from all participants before the experiments were conducted. All participants reported normal or corrected-to-normal vision, hearing, and somatosensation and no neurological abnormalities.

->

They were recruited randomly from an introductory psychology class, and written informed consent was obtained from all participants before the experiments were conducted. None of participants had abnormalities in vision, hearing, and somatosensation.

**P9, left column**

L10

The most discriminating tactile sensors among primates - the fingertips - house a few hundred sensory nerve fibers per square cm (Johansson and Vallbo, 1979; Darian-Smith and Kenins, 1980).

->

The fingertips, where in primates the most discriminating tactile sensors are located, have a few hundred sensory nerve fibers per square centimeter (Johansson and Vallbo, 1979; Darian-Smith and Kenins, 1980).

L34

A typical tau effect, where the perceived distance between stimuli underestimates, and grows in proportional with, the actual distance when stimulus timing is held constant (Marks et al.,1982; Cholewiak, 1999) and the kappa effect, in which the perceived time between stimuli dilates as the distance between stimuli is increased (Suto, 1952), reflect just two fundamental perceptual distortions: underestimation of inter-stimulus distance (perceptual length contraction) and overestimation of inter-stimulus time interval (perceptual time dilation; Goldreich, 2007).

->

A typical tau effect, where the perceived distance between two stimuli gets longer or shorter depending on the actual inter-stimulus distance when stimulus timing is held constant (Marks et al.,1982; Cholewiak, 1999), and the kappa effect, in which the perceived time between stimuli increases when the stimuli distance gets longer (Suto, 1952), reflect “just two fundamental perceptual distortions”: underestimation of inter-stimulus distance (perceptual length contraction) and overestimation of inter-stimulus time interval (perceptual time dilation; Goldreich, 2007).

L42

Given that three successive tactile stimuli define two spatial (S1 and S2) and two temporal intervals (T1 and T2), the somatosensory system intuitively imputes motion at a given speed to the tactiles and tries to equalize the ratios S1/S2 and T1/T2; thus, it follows that S1/T1 = S2/T2 (modified from Jones and Huang, 1982).

->

Two spatial distances (S1 and S2) and two temporal intervals (T1 and T2) are defined by three successive tactile stimuli. The somatosensory system intuitively interprets these stimuli as motion at a certain speed, equalizing the ratios S1/S2 and T1/T2; thus, S1/T1 = S2/T2 (modified from Jones and Huang, 1982).

L47

In this way, the sensory system - which includes somatosensation, vision, and audition (Cohen et al., 1953; Shore et al., 1998) - attempts to equalize the velocity between the first and second stimuli (S1/T1) and that between the second and third stimuli (S2/T2); this is known as the constant velocity assumption (Goldreich, 2007).

->

In this way, the sensory system - which includes somatosensation, vision, and audition (Cohen et al., 1953; Shore et al., 1998) - tries to equalize the velocity between the ratios S1/T1 and S2/T2; which is the constant velocity assumption (Goldreich, 2007).

**P9, right column**

L9

Thus, the general tendency is to displace the judged position of a moving target as being relatively far forward along the path of motion (Tremoulet and Feldman,2006; Getzmann and Lewald, 2007, 2009).

->

Thus, the general tendency is that the judged position of a moving object is displaced forward along the direction of motion (Tremoulet and Feldman,2006; Getzmann and Lewald, 2007, 2009).

L31

When participants discriminate the locations of vibrotactile stimuli by ignoring distractor lights, such tactile discriminations are slowed when the distractor light is incongruent with the tactile target (Pavani et al., 2000).

->

When participants discriminate the locations of vibrotactile stimuli by ignoring distractor lights, the reaction time in tactile discriminations is delayed when the distractor light is spatially incongruent with the tactile stimuli (Pavani et al., 2000).

L39

The rubber hand illusion (RHI) refers to the effect of watching a rubber hand being stroked synchronously with one's own, unseen hand. Viewing this for a short time causes the observer to perceptually assimilate the rubber hand into his or her own body (Botvinick and Cohen, 1998).

->

The rubber hand illusion (RHI) is a tactile illusion where watching a rubber hand being stroked synchronously with one's own hand for a short time causes the subjective feeling of illusory body-ownership of the rubber hand or causes the sense of attribution of the rubber hand to participants' own body (Botvinick and Cohen, 1998).

**P10, left column**

L14

Cross-modal causality plays a key role in governing the integration of sensory information, depending on its ecological plausibility (Schutz and Kubovy, 2009).

->

Causality perception among modalities is fundamental for integrating sensory information, depending on the ecological plausibility (Schutz and Kubovy, 2009).

L17

Humans can use the similarities between the temporal structures of sensory signals in different modalities to solve the correspondence problem, ultimately inferring causation from correlation (Parise et al., 2012).

->

Humans can use the similarities between the temporal structures of sensory signals in different modalities in order to handle the correspondence problem, “ultimately inferring causation from correlation” (Parise et al., 2012).

L30

Illusory sequences activate the contralateral primary somatosensory cortex at somatotopic locations corresponding to the filled-in illusory perceptions on the forearm (Blankenburg et al., 2006); this suggests that this illusion is associated with the early sensory body map represented in S1.

->

Illusory sequences activate the contralateral primary somatosensory cortex (Blankenburg et al., 2006), suggesting an association with the early sensory body map in S1.

L38

Small amounts of latency between vision and touch (or sound) tend to be reduced and go unnoticed, because the timing of visual events is flexible and adjusts immediately (for a review, see Vroomen and Keetels,2010).

->

Small amounts of latency between vision and touch (or sound) tend to be reduced because the timing perception in vision is flexible and adjusts immediately (for a review, see Vroomen and Keetels,2010).

L53

The CRE and its interaction with vision indicate that sensory events at a given time point are influenced by future sensory events; this is called “postdictive” sensation (Eagleman and Sejnowski, 2000).

->

The CRE and its interaction with vision indicate that future sensory events, even if the modalities are crossed, affect past sensory experiences. This is called “postdictive” sensation (Eagleman and Sejnowski, 2000).

